# Inbreeding Depression Manifested in Progeny From Fragmented Populations of the Wind‐Pollinated Dioecious Conifer *Afrocarpus gracilior* (Pilg.) C. N. Page

**DOI:** 10.1002/ece3.70903

**Published:** 2025-02-12

**Authors:** Nigussu Begashaw Abate, Hewan Demissie Degu, Marie Kalousová, Tesfaye Abebe

**Affiliations:** ^1^ School of Plant and Horticultural Sciences, College of Agriculture Hawassa University Hawassa Ethiopia; ^2^ Department of Crop Sciences and Agroforestry, Faculty of Tropical Agrisciences Czech University of Life Sciences Prague Prague Czech Republic

**Keywords:** gene flow, genetic diversity, in vitro germination, inbreeding, progeny fitness

## Abstract

Human activities such as agriculture, urbanization, and logging have caused widespread destruction of forests, leading to forest fragmentation. Fragmentation has been shown to reduce genetic diversity and increase inbreeding in forest populations, potentially leading to inbreeding depression manifested through decreased reproductive success and progeny vigor. The severity of these impacts, however, varies among species and is largely influenced by their mating systems. This study examines the effects of forest fragmentation on *Afrocarpus gracilior*, a dioecious, wind‐pollinated conifer, by assessing genetic diversity, reproductive success, and early progeny fitness. Our analysis revealed alarmingly low genetic diversity and high genetic drift, especially in small and isolated populations. Consistent with these findings, reduced progeny fitness was observed, with small populations showing 53% lower germination rates, 33% reduced acclimatization, 30% slower diameter growth, 41% reduced height growth, and an 80% increase in leaf scorch. Correlation analysis further confirmed a strong relationship between the genetic diversity and progeny fitness traits. These findings suggest that inbreeding depression severely affects the fitness of progeny from small and isolated populations of *A. gracilior* , posing a serious threat to their long‐term survival. The implications for conservation and restoration efforts are immense, underscoring the need to prioritize genetically diverse populations for conservation and strategically procure seeds to support the survival of this species.

## Graphical abstract

Forest fragmentation in *Afrocarpus gracilior* reduced genetic diversity and caused significant inbreeding depression, especially in smaller populations. Progeny from these populations showed lower genetic diversity, reduced germination rates, slower growth, and diminished survival. Seeds should be sourced from larger, genetically diverse populations for effective conservation and restoration.

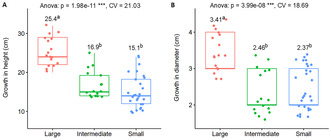

## 1 Introduction

Human activities such as agriculture, urbanization, and logging have caused widespread destruction of forests, leading to forest fragmentation. Fragmentation occurs when large, intact, and continuous forests are broken into smaller, isolated patches separated by nonforest land (Young and Boyle 2000; Bacles and Jump 2011; Schlaepfer et al. 2018). In these fragmented forest patches, the reduction in population size and increased isolation limits the number of available pollen donors and restricts pollinator movement. This results in a decrease in both the quantity and diversity of pollen being deposited (Cunningham 2000; O'Connell, Mosseler, and Rajora 2006; Seltmann et al. 2007; Broadhurst 2015). The restricted gene flow also leads to mating between related individuals and self‐pollination (in self‐compatible species), which causes inbreeding, a loss of genetic diversity, and genetic drift (Ellstrand and Elam 1993; Aguilar and Galetto 2004; Bacles and Jump 2011; Lloyd, Tumas, and Neel 2018). The reduction in genetic diversity and the prevalence of inbreeding diminishes a population's ability to adapt to environmental changes. This is evidenced by reduced progeny fitness, known as inbreeding depression, which results from the expression of deleterious mutations in homozygous inbred offspring (Charlesworth and Willis 2009; Eckert et al. 2010). Inbreeding depression is manifested through decreased reproductive success and lower progeny vigor (Cascante et al. 2002; Seltmann et al. 2007; Aguilar et al. 2019; Aguilar‐Aguilar et al. 2023), which can ultimately threaten the long‐term survival of populations and potentially lead to local extinction (Young, Boyle, and Brown 1996; Frankham 2005; Reed 2005; Schlaepfer et al. 2018; Phang et al. 2024).

Over the past three decades, the genetic and demographic impacts of forest fragmentation have been the focus of extensive research, supported by advancements in molecular marker technologies (reviewed by Young, Boyle, and Brown 1996; Lowe et al. 2005; Kramer et al. 2008; Vranckx et al. 2012; Finger et al. 2014; Schlaepfer et al. 2018; Aguilar et al. 2019; González et al. 2020). The findings of these studies have been diverse. Some have demonstrated that fragmentation negatively impacts genetic diversity and progeny fitness in trees (e.g., Cascante et al. 2002; Aguilar and Galetto 2004; Hirayama, Ishida, and Tomaru 2005; Jump and Peñuelas 2006; Seltmann et al. 2009; Lloyd, Tumas, and Neel 2018; Aguilar‐Aguilar et al. 2023; Phang et al. 2024). For instance, Cascante et al. (2002) found that seedlings of *Samanea saman* from isolated trees exhibited lower germination rates, reduced leaf area, and biomass compared to those from continuous forest populations. Similarly, Seltmann et al. (2009) reported reduced vigor (lower N‐metabolism capacity) and high mortality in *Polylepis australis* seedlings from short‐distance pollination crosses compared to those from long‐distance crosses. Lloyd, Tumas, and Neel (2018) highlighted limited pollen dispersal distances and biparental inbreeding in *Vallisneria americana* , exacerbated by habitat fragmentation. Recently, Phang et al. (2024) identified genetic erosion, marked by reduced heterozygosity and increased inbreeding in juvenile populations compared to adult populations of *Palaquium obovatum*.

In contrast, other studies suggest that gene flow in some tree species remains largely unaffected by habitat fragmentation, indicating potential resilience (e.g., White, Boshier, and Powell 2002; O'Connell, Mosseler, and Rajora 2007; Silva et al. 2008; Kamm et al. 2009; Lander, Boshier, and Harris 2010; Ashworth et al. 2015; Broadhurst 2015; Guidugli et al. 2016). This resilience, against the genetic expectations for small and fragmented populations, has been described as “the paradox of fragmentation genetics” (Kramer et al. 2008). However, Lowe et al. (2015) argued that this phenomenon is “no longer a paradox,” emphasizing that earlier studies primarily focused on adult populations, which often fail to capture current gene flow dynamics in long‐lived trees. They described this narrow focus as “looking in the wrong place” and called for more comprehensive approaches. In response, recent research has adopted a broader approach, incorporating both adult and progeny cohorts in genetic diversity assessments, conducting gene flow and paternity analyses, and examining diverse mating systems and pollination mechanisms. This shift has provided deeper insights into the interplay between forest fragmentation and genetic diversity, offering a more nuanced understanding of tree species' responses to environmental changes.

A common shift in mating systems involves reduced outcrossing and increased selfing, which may result in inbreeding depression (Aguilar et al. 2006; Broadhurst 2015). Inbreeding depression typically results in reduced seed production and less vigorous progeny. While most tropical trees are primarily outcrossing (Cascante et al. 2002; Ward et al. 2005), their hermaphroditic and self‐compatible nature makes them facultative out‐crossers (Eckert et al. 2010). In fragmented habitats, a reduction in the number of mating trees disrupts pollinator movement, hindering effective pollination (Wilcock and Neiland 2002; Breed et al. 2015). Pollinators adapt by shortening their foraging distances and spending more time on individual trees (Breed et al. 2015). Consequently, trees dependent on these pollinators are forced to lower their outcrossing rates and increase selfing (Aguilar et al. 2006; Breed et al. 2015; Broadhurst 2015; Aguilar et al. 2019). According to Aguilar et al. (2019), the severity of inbreeding depression due to selfing varies with a species' mating history. Populations with a long history of selfing are less prone to severe inbreeding depression, as continuous selfing over generations tends to purge deleterious alleles. In contrast, species that predominantly outcross but shift to selfing due to habitat fragmentation are likely to suffer significant inbreeding depression, particularly in early fitness traits such as germination and survival.

Dioecious and self‐incompatible species are obligate out‐crossers, meaning that they cannot shift to selfing (Aguilar et al. 2006; Broadhurst 2015; Cristóbal‐Pérez et al. 2021; Aguilar‐Aguilar et al. 2023). Dioecism serves as an adaptation to prevent self‐fertilization and enhance heterozygosity by involving multiple parents in reproduction (Arruda et al. 2015). Consequently, selfing‐induced inbreeding is inherently impossible. Nonetheless, biparental inbreeding can still occur, involving mating between closely related individuals (Broadhurst 2015; Arruda et al. 2015; Vinson et al. 2018). Dioecious species are particularly susceptible to the effects of habitat fragmentation (Aguilar et al. 2006; Cristóbal‐Pérez et al. 2021; Aguilar‐Aguilar et al. 2023; Aguilar et al. 2024), as decreases in population size and density can influence several ecological factors that affect reproductive success. These factors include the sex ratio, the spatial distribution of male and female trees, and the foraging behavior of pollinators.

Dioecious species typically rely on small insects and wind for pollination (Cristóbal‐Pérez et al. 2021; Aguilar‐Aguilar et al. 2023). Habitat fragmentation can restrict the mobility of small insect pollinators, making long‐distance foraging inefficient (Breed et al. 2015), which reduces pollen availability for female dioecious trees. This pollen limitation often results in lower seed set (Wilcock and Neiland 2002; Ohya, Nanami, and Itoh 2017) and may ultimately disrupt reproduction in fragmented populations. In contrast, wind pollination is commonly expected to facilitate long‐distance gene flow (Seltmann et al. 2007; Provan et al. 2008; Ashley 2010, 2021; Dubreuil et al. 2010; Broadhurst 2015; Zeng and Fischer 2019; Aguilar‐Aguilar et al. 2023), potentially reducing the vulnerability of wind‐pollinated species to the genetic impacts of fragmentation.

Nonetheless, evidence suggests that even wind‐pollinated species are not immune to fragmentation‐induced genetic challenges, with several studies documenting declines in reproductive success and progeny fitness due to inbreeding. Examples include *Quercus douglasii* (Knapp, Goedde, and Rice 2001), *Fagus sylvatica* (Jump and Peñuelas 2006), *Picea glauca* (O'Connell, Mosseler, and Rajora 2006), *Araucaria nemorosa* (Kettle et al. 2007, 2008), *Juniperus communis* (Provan et al. 2008), *Polylepis australis* (Seltmann et al. 2009), *Taxus baccata* (Dubreuil et al. 2010), and *Brosimum alicastrum* (Aguilar‐Aguilar et al. 2023). For instance, Jump and Peñuelas (2006) demonstrated that habitat fragmentation in *Fagus sylvatica* caused genetic bottlenecks, disrupted breeding systems, and significantly elevated inbreeding, population divergence, and reduced genetic diversity—even in this widely distributed, wind‐pollinated species. Similarly, Aguilar‐Aguilar et al. (2023) found that fragmentation altered mating patterns in *Brosimum alicastrum* , a dioecious, wind‐pollinated tree, resulting in progeny with reduced fitness, evidenced by fewer leaves with smaller foliar areas, shorter heights, and lower biomass compared to progeny from continuous forests. These mixed findings suggest that the impact of fragmentation on wind‐pollinated species is complex and species‐specific, underscoring the need for further research to better understand the genetic and ecological dynamics of trees in fragmented landscapes.

**FIGURE 1:**
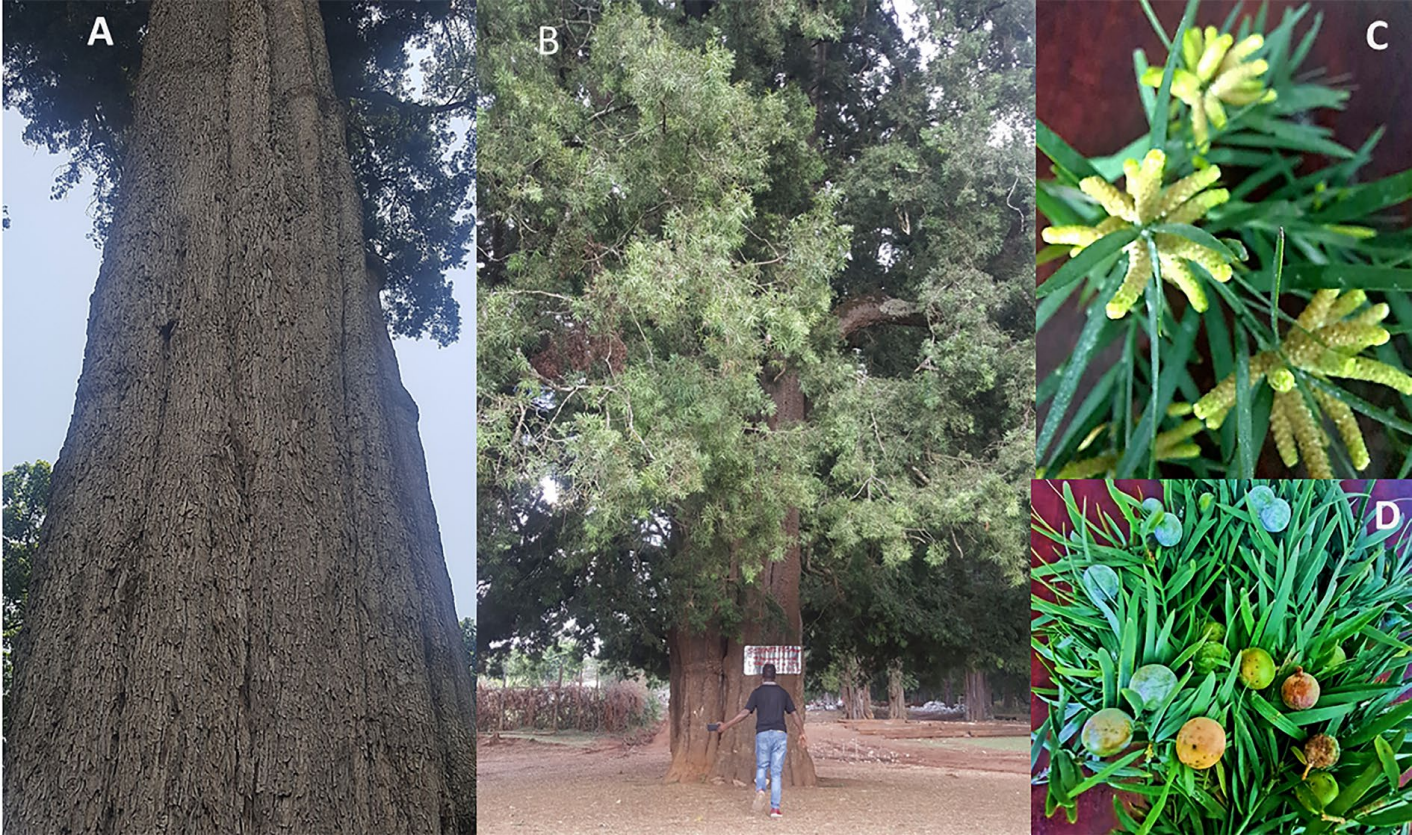
Photographs of *Afrocarpus gracilior*. (A) The trunk of a tall, mature *A. gracilior* tree, which makes it a valuable timber species; (B) A large *A. gracilior* tree with a partial view of its crown; (C) Male cones of *A. gracilior* , showing spirally arranged sporophylls responsible for releasing pollen grains; (D) Female cones or “fruits” of *A. gracilior* with their conspicuous fleshy outer covering called the epimatium; the cones are green when unripe and turn yellowish upon ripening.

In this study, we explored the effects of fragmentation on the reproductive success and early progeny fitness of *Afrocarpus gracilior* (Pilg.) C. N. Page, a member of the Podocarpaceae family. Also ambiguously referred to as *Podocarpus falcatus* or *Podocarpus gracilior*, the species is known by several common names such as East African yellowwood, African fern pine, or weeping Podocarpus. This wind‐pollinated, dioecious conifer (Figure 1) is native to Ethiopia and other parts of East and Central Africa. Once dominant in the canopy of Afromontane forests across Ethiopia, *A. gracilior* has long been threatened by illegal logging for its high‐quality timber (Negash 2010; Teketay 2011). Today, it is primarily found in inaccessible relict forest patches (Negash 2003, 2010), some church forests (Aerts et al. 2016), and other culturally sacred sites (Doffana 2014; Doda and Abuelgasim 2019). Although classified as ‘Least Concern’ on the IUCN Red List due to its wide distribution, including areas outside Ethiopia, *A. gracilior* is considered threatened within Ethiopia and is among the few native trees prioritized for conservation efforts in the country (Vivero, Kelbessa, and Demissew 2005; Kalinganire, Moestrup, and Graudal 2021). In the Sidama and Gurage regions of southern Ethiopia, however, *A. gracilior* remains relatively abundant in various fragmented populations, such as isolated individuals, small groups in front yards, graveyards, sacred sites, and natural forest remnants. The impact of fragmentation on the genetic diversity of these populations and the long‐term viability of offspring from their seeds has not yet been examined.

A companion study (Abate et al. 2024) assessed genetic diversity, using DArTseq‐generated SNP markers, in adult and progeny cohorts from fragmented populations of varying sizes. This study revealed very low genetic diversity and high genetic drift, especially in progeny from isolated and small populations. The overall mean expected heterozygosity (He) was 0.072, and the mean allelic richness (Ar) was 1.24, with the maximum for SNP markers (which are biallelic) being 2. The mean He and Ar values for the adult populations were 0.075 (range: 0.067–0.081) and 1.243 (range: 1.218–1.278), respectively, while for the progeny populations, these values were 0.069 (range: 0.055–0.076) and 1.229 (range: 1.19–1.257), respectively. For both He and Ar, smaller scores were recorded in small/isolated populations, and larger scores were recorded in relatively larger/intact populations. Similarly, progeny in small/isolated populations exhibited higher population‐specific *F*st values indicating significant drift from ancestral populations. Building on these findings, the present study aims to explore whether the genetic erosion observed at the molecular level manifests as inbreeding depression, characterized by reduced reproduction and progeny fitness. Specifically, this study seeks to (1) determine whether populations of different sizes differ in terms of physical seed quality traits; (2) evaluate differences in germination rates among seeds from these populations; and (3) assess variation in seedling survival and early progeny vigor. These insights are crucial for the conservation and restoration of *A. gracilior* in Ethiopia and other regions where the species is native.

## 2 Materials and Methods

### 2.1 Description of the Seed Source Populations

**FIGURE 2:**
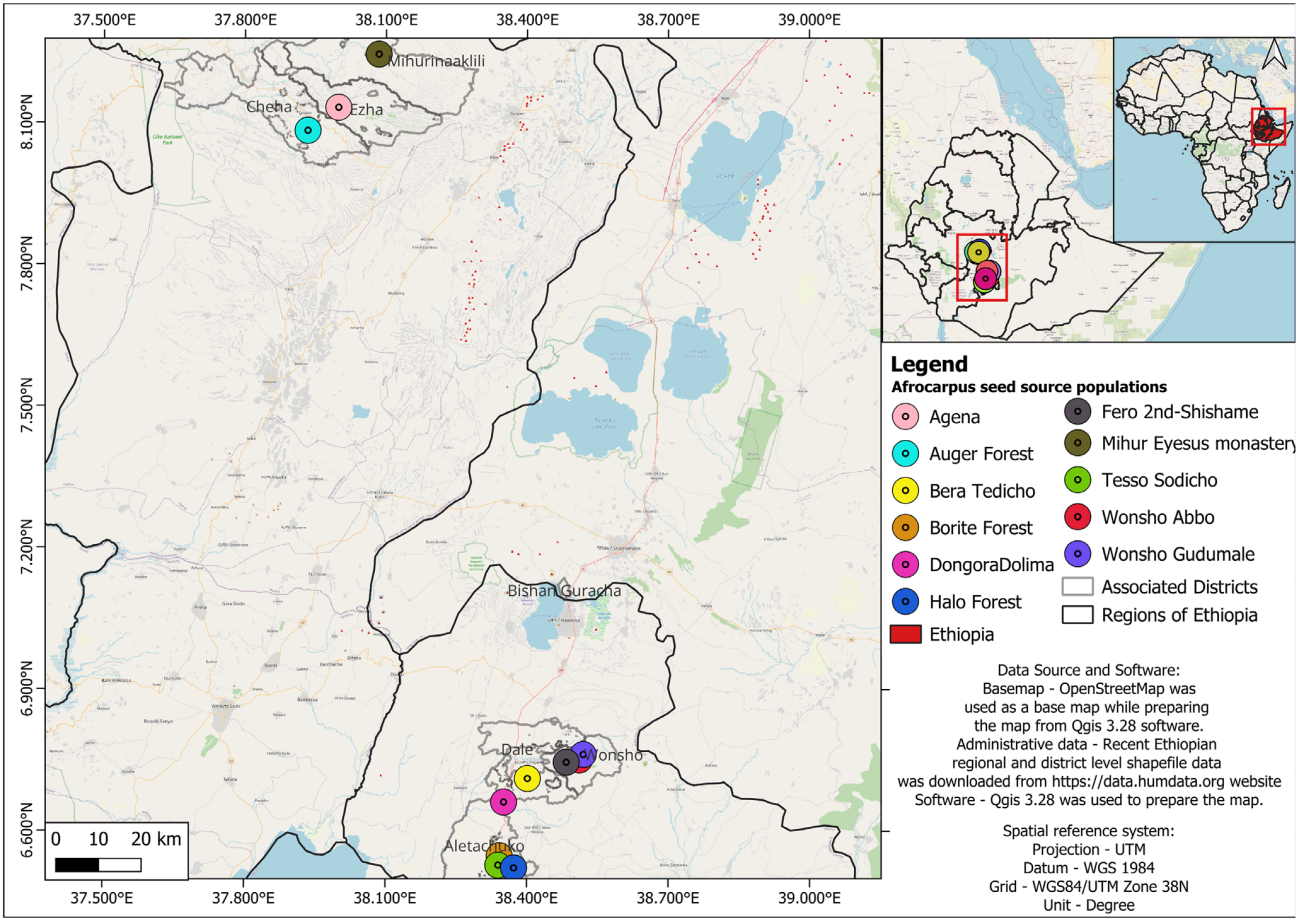
Map of Ethiopia showing the locations of the sampling populations of *A. gracilior* . Southern populations represent the Sidama region, whereas those in the north belong to Gurage.

**FIGURE 3:**
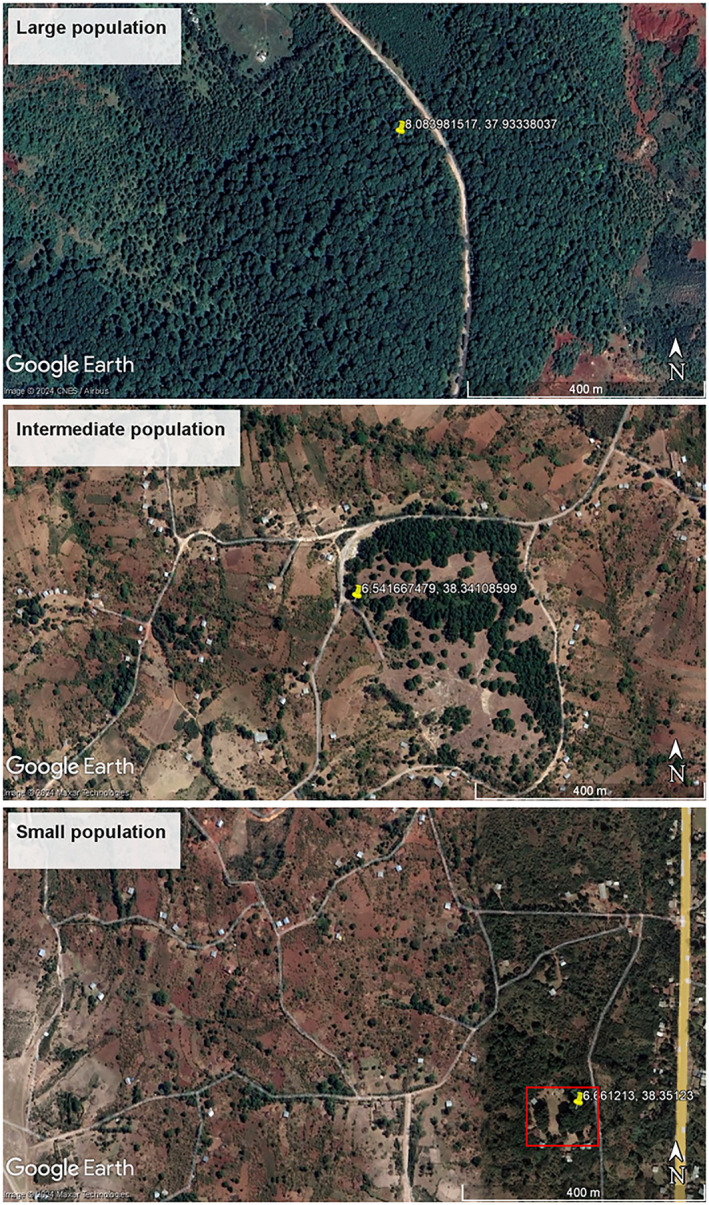
Google Earth images displaying examples of the different population types of *A. gracilior* included in this study. From top to down, there is a relatively large and intact remnant of an *A. gracilior* ‐dominated natural forest patch in Gurage (Auger in Table 1), an intermediate open natural remnant patch in Sidama (Borite in Table 1), and a small population consisting of a few *A. gracilior* trees at a communal graveyard, highlighted by the box (Dangora–Dolma in Table 1).

Seeds of *A. gracilior* were collected from selected populations in the Sidama and Gurage regions of southern Ethiopia (Figure 2). In these regions, the size distribution of *A. gracilior* populations varies significantly. Isolated trees or small groups of fewer than 10 individuals are commonly found in front yards, whereas groups of 10–50 individuals occur in graveyards and communal gathering places, known locally as *gudumales*. Conversely, remnant natural forest patches and sacred sites, such as churches and traditional worship places, host relatively large populations (Table 1, Figure 3). Populations for this study were chosen following a reconnaissance survey, ensuring representation from all population types. On the basis of their size, populations were categorized as ‘small’ (area < 1 ha), consisting of isolated individuals or groups not more than approximately 50 trees; ‘intermediate’ (1 ≤ area ≤ 50 ha); and ‘large’ (area > 50 ha).

**TABLE 1:** Description of the *A. gracilior* populations examined in this study. The Agena, Fero 2nd‐Shishame, Dongora‐Dolima, and Halo Forest populations were not included in the genetic diversity analysis; they were only part of the reproduction and early progeny vigor assessments. Consequently, data from these populations are not included in the genetic diversity summary (Table 2) or the correlation analysis (Figure 10).

Site/population	Region/zone	Altitude (m a.s.l.)	Population size class	Description
Agena	Gurage	2344	Small/isolated	An old female tree situated along a roadside in a town stands isolated, with no nearby conspecifics to act as pollen donors. Although seed production is relatively high, there is no observed regeneration beneath its crown
Auger forest	Gurage	2226	Large/intact	A remnant natural forest patch featuring a mixed Afrocarpus–Juniperus community is relatively large and intact, covering 138 ha with a density of approximately 250 trees per hectare. Although many trees produce seeds, the seed output per tree is relatively low. Nevertheless, there is healthy regeneration beneath the canopy
Bera‐Tedicho	Sidama	1807	Small/isolated	A small group of old remnant trees in a village comprises 54 trees within a 0.5‐ha area. The canopy is open, and seed production per tree is relatively high. While no regeneration is observed under the canopy, it is observed in nearby farms
Mihur Eyesus monastery	Gurage	2330	Intermediate	This old monastery is situated on a plateau, featuring a front yard with several ancient *A. gracilior* trees, some over 200 years old. The plateau gradually slopes down into a dense, mixed‐species forest spanning 16 ha, with an *A. gracilior* density of approximately 100 trees per hectare. The forest exhibits a range of tree sizes, from juveniles to intermediate‐sized trees, indicating robust regeneration. While the few ancient trees in the monastery's front yard produce a high number of seeds per tree, seed production is minimal in the dense canopy of the forest below
Borite forest	Sidama	1930	Intermediate	This small natural remnant patch, located on a hilltop and covering about 10 ha, has dense regeneration with numerous juvenile trees but relatively few adults (about 75 trees per hectare). The forest features an open canopy and exhibits relatively high seed production per tree
Fero 2nd‐Shishame	Sidama	1903	Small/isolated	wo old trees, one male and one female, are situated at the edge of a 0.5‐ha communal front yard. These trees are isolated, with no other conspecifics visible nearby. The female tree produces a substantial number of seeds. No regeneration beneath canopy
Dongora‐Dolima	Sidama	1800	Small/isolated	This communal graveyard, situated in the front yards of several houses, spans about 1 ha and contains 53 adult trees, some of which are quite old. While regeneration is not observed beneath the canopy, it is present along fences by the road and in adjacent home gardens. The trees are widely spaced, and each tree produces a relatively high amount of seeds
Halo forest	Sidama	1974	Intermediate	This segment of a relatively large natural remnant forest, situated on a continuous hilltop bordering three kebeles (rural counties), is facing encroachment due to farmland expansion and illegal timber harvesting. Seeds of *A. gracilior* were collected from the foothill on the Dara side of the forest, which covers about 6 ha. The tree density here is sparse, approximately 100 trees per hectare. Regeneration is good at some spots, with a variety of age classes present. The canopy, dominated by a few old trees, is open, and seed production per tree is relatively high
Tesso‐Sodicho	Sidama	1795	Small/isolated	In the front yards of houses in a village, a few adult *A. gracilior* trees are present. A single isolated female tree, located over 200 m from the nearest male trees, is the primary seed bearer in this area. This tree produces a very high quantity of seeds. Regeneration has been observed along fences and in adjacent home gardens
Wonsho‐Abbo	Sidama	2060	Large/intact	This population occupies the lower half of a culturally protected worship and traditional court site. The site encompasses 75 ha of mixed forest with numerous indigenous tree species. The lower segment, predominantly composed of *A. gracilior* , is relatively dense, with 250 trees per hectare, and exhibits good regeneration. However, the seed production per tree is low
Wonsho‐Gudumale	Sidama	2110	Small/isolated	The site features 30 very large and old trees, some over 200 years old. This area, used for centuries as a communal gathering space known locally as a *gudumale* for cultural events, spans approximately 0.5 ha. Only a few of these trees are seed‐bearing, producing a high quantity of seeds. While there is no regeneration beneath the canopy, some regeneration is observed in the adjoining fences and home gardens

### 2.2 Genetic Diversity

Detailed methods for the genetic diversity analysis, including sampling, genotyping, and data processing, are outlined in the companion paper (Abate et al. 2024). Briefly, young leaf tissue samples were collected from adult and progeny populations of *A. gracilior* across 11 sites in southern Ethiopia. Two plates (188 samples) of silica‐dried leaf tissues were sent to SEQART Africa in Kenya for DNA extraction and genotyping. DNA was extracted using the CTAB‐based Nucleomag Plant Extraction Kit (Macherey‐Nagel, Düren, Germany) and genotyping was conducted using DArTseq technology, which integrates a proprietary genome complexity reduction method with next‐generation sequencing. The genotyping yielded two types of markers: silicoDArT markers, which are dominant and scored as binary, and SNP markers, which are biallelic and codominant. For the genetic diversity analyses, only the SNP markers were utilized due to their higher informativeness for population genetics studies compared to the dominant silicoDArT markers. After rigorous filtering of the raw SNP dataset, which initially contained 10,219 SNPs and 185 individuals, 1820 high‐quality, informative SNPs, and 183 individuals were retained for downstream analysis.

### 2.3 Population Inventory and Seed Collection

Inventories were conducted on the selected populations to determine population sizes, tree density, size‐class distribution, estimated age of the dominant trees, and seedling recruitment (availability and density of seedlings and saplings under the canopy of adult trees). For the small populations, the inventory was based on a total census. For the intermediate and large populations, we used 10–15 randomly distributed 10‐m radius circular plots. Observations on seedling recruitment and density observations were carried out using four 1 m × 1 m quadrats nested within each sampling plot. Since *A. gracilior* is a dioecious species, an assessment was also made on the availability of male individuals near female individuals to ensure adequate pollination. Seed setting by female trees (those bearing female cones) was observed, and estimates of seed yield were noted. Mature female cones were collected both from the crowns and those that had fallen to the ground. In smaller populations, cones were collected from all cone‐bearing female trees, while in larger populations, cones from 15 to 20 female trees were collected, depending on availability. Equal amounts of cones collected from each tree in each population were then combined into a single bag representing that population.

**FIGURE 4:**
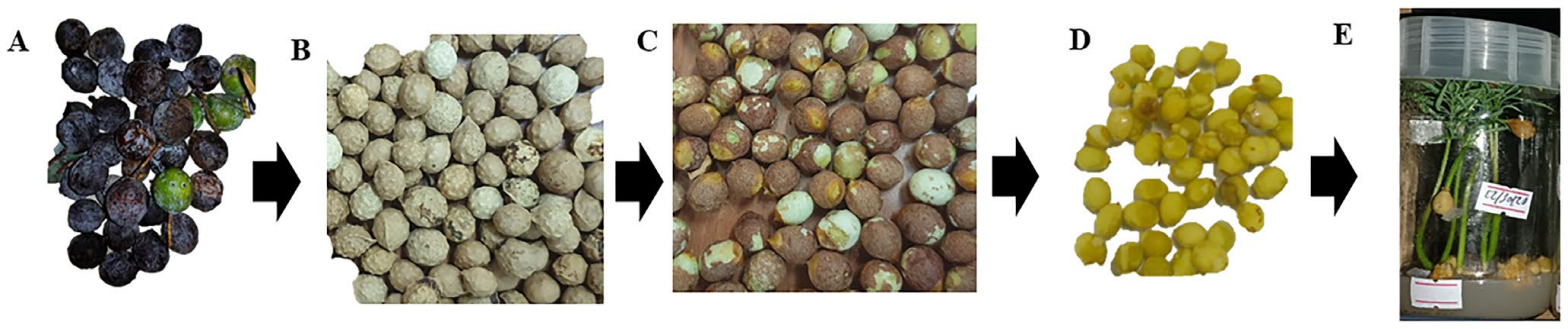
Process of *A. gracilior* seed collection to in vitro germination. (A) Ripe female cones after collection; (B) seeds showing the hard seed coat (sclerotesta) after removal of the fleshy pulp (epimatium); (C) released female gametophytes (true seeds) after cracking the sclerotesta; (D) surface sterilized seeds ready for in vitro germination; and (E) in vitro germinated *A. gracilior* seedlings in a tissue culture jar.

Mature female cones collected in this manner were then transported to the forest seed laboratory of the College of Agriculture at Hawassa University. Seed extraction was performed following the procedure outlined in Negash (2010). Briefly, the process involved soaking the cones in water for 2 days, gently squashing them with a wooden mortar and pestle, and washing them to remove the fleshy pulp from the inner seeds (Figure 4). The de‐pulped seeds, which still had a hard seed coat or sclerotesta, were air‐dried in the shade for 2–3 days, placed in seed bags, and stored in a refrigerator at 4°C until they were used for subsequent in vitro germination.

### 2.4 Physical Seed Quality Assessment and *In Vitro* Germination

The sclerotesta of an *A. gracilior* seed is a hard ‘woody’ structure that covers and protects the megagametophyte, also known as the female gametophyte or the true seed (Negash 2010). For germination to occur, the sclerotesta has to be cracked to release the female gametophyte (Figure 4B,C). When the sclerotesta is cracked, clean and intact seeds may be released, the seeds may be damaged (either immature and shriveled or infested by microorganisms), or they may be empty altogether. In this study, we used the proportions of intact, empty, and damaged seeds, along with seed weight, as measures of physical seed quality. We randomly selected 100 seeds (5 replicates of 20 seeds each), cracked them, counted the number of intact, empty, and damaged seeds, and determined the percentage proportion of each. After that, we took 100 intact seeds (5 replicates of 20 seeds each), measured their weight on a digital balance, and calculated 1000 seed weights.

Prior to in vitro germination, the seeds (female gametophytes) were surface sterilized by soaking in a solution of 5% sodium hypochlorite followed by 70% ethanol for 10 min each. The seeds were subsequently rinsed in distilled and sterilized water three times to remove disinfectants. The disinfected seeds were then placed in a 10^−4^ M solution of gibberellic acid‐3 (GA_3_) to stimulate germination. Seeds recovered from this solution (Figure 4D) were placed in tissue culture jars (Figure 4E), with 5 replicates of 10 seeds per jar, containing 50 mL of 20 g/L melted and autoclaved agar. The jars were kept in the culture room of the tissue culture laboratory at Hawassa University, College of Agriculture in a completely randomized design (CRD). The room was maintained with 16 h of light from fluorescent lamps, providing a photon fluorescence rate of 100 μmol m^−2^ s^−1^ at a temperature of 25°C. The in vitro germination rate was monitored, and data recording was performed every 3 days over a 10‐week experimental period.

### 2.5 Assessment of Early Progeny Vigor in a Lathhouse

**FIGURE 5:**
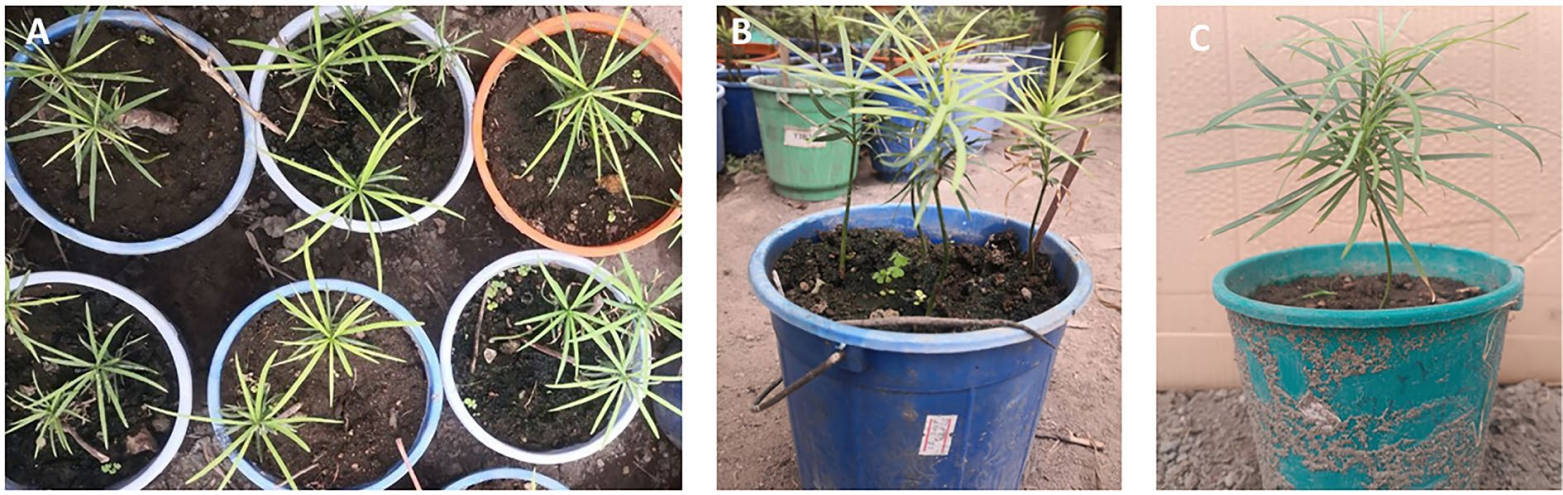
Acclimatization of in vitro germinated seedlings in the lathhouse: (A) top view of 2‐month seedlings in a group of pots; (B) a closer view at a single pot; and (C) a single seedling per pot at the end of the experiment.

At the end of the 10‐week experiment in the tissue culture lab, the in vitro germinated seedlings were transferred to a lathhouse for acclimatization and monitoring of early progeny vigor. The lathhouse was a shade house covered with a plastic roof and mesh wire ventilation. The seedlings were grown in 3‐L plastic pots filled with mixed growing media composed of local agricultural soil, compost and sand at a 2:1:1 ratio, and arranged in a CRD. Initially, all in vitro germinated seedlings in a single jar were transferred to a single pot. Later, after acclimatization, the seedlings were singled out and transferred to other pots so that only a single seedling would grow in a single pot (Figure 5).

The survival of the seedlings in the lathhouse environment (i.e., their acclimatization) was assessed 1 month after they were transferred. Monitoring of the seedlings continued until the end of the 8‐month experiment, with periodic measurements of seedling growth in terms of root collar diameter (using a Vernier caliper) and height (using a graduated ruler) taken. Additionally, the percentage of leaf scorch (of each plant) was estimated as an indicator of susceptibility to heat stress in the lathhouse. Given that the temperature in the lathhouse occasionally becomes very hot (reaching over 30°C), this was considered a coincidental stress treatment against which the susceptibility of the seedlings was evaluated. Acclimatization and leaf scorch scores were taken as measures of the survival/mortality of the seedlings in the lathhouse environment.

### 2.6 Data Analysis

A detailed analysis of genetic diversity, population structure, and phylogenetic relationships is provided in the companion study (Abate et al. 2024). For this study, key genetic diversity metrics from that study—allelic richness (Ar), unbiased expected heterozygosity (uHe), and population‐specific *F*st—were summarized to link genetic patterns with the seed quality and progeny fitness results of the present study. Expected heterozygosity (He), also known as Nei's gene diversity, reflects the average proportion of heterozygotes expected per locus under Hardy–Weinberg equilibrium and is a widely recognized metric for assessing genetic diversity (Nei 1973; Harris and DeGiorgio 2017; Barrandeguy and García 2021). Unbiased expected heterozygosity (uHe) adjusts for biases from small sample sizes, ensuring more accurate comparisons across populations (Harris and DeGiorgio 2017). Allelic richness (Ar), which measures the number of alleles per locus and can be standardized using rarefaction for populations of varying sizes, is particularly valuable for conservation prioritization (Petit, Mousadik, and Pons 1998; Barrandeguy and García 2021). While both He and Ar are sensitive to shifts in allele frequency, Ar is more responsive in long‐lived trees and self‐incompatible species (González et al. 2020; Barrandeguy and García 2021). The inbreeding coefficient (*F*is), calculated from discrepancies between observed and expected heterozygosity, is another common parameter. However, it is often less sensitive to habitat fragmentation (Schlaepfer et al. 2018) and is considered less reliable as a standalone measure of inbreeding (Kardos et al. 2016). Population‐specific *F*st, a measure of the probability of two alleles being identical by descent within a population relative to alleles from different populations, has been suggested as a more robust indicator of inbreeding and genetic drift than *F*is (Weir and Goudet 2017; Kitada et al. 2021; Abate et al. 2024).

The data from the physical seed quality assessment, in vitro germination, acclimatization in a lathhouse, and early progeny vigor assessment were subjected to one‐way analysis of variance (ANOVA), with populations grouped by population size classes taken as independent variables. For the in vitro germination analysis, a one‐way ANOVA was performed on the final germination data at the end of the 10‐week experiment. The cumulative germination data, collected every 3 days, were plotted in line graphs with error bars to indicate significant differences between groups, illustrating the germination trend over the test period. The ANOVA models were fitted in R4.3.3 statistical software (R Core Team 2024) via the *aov* function. The results of the models were visualized via the *summary* function, and variances were considered significant when the *p* values were less than 0.05. The assumptions of homoscedasticity (i.e., constancy of variance) and a normal distribution were checked by plotting the models in R via the *plot* function and visualizing the standardized versus fitted and normal Q–Q plots of the residuals (Schützenmeister, Jensen, and Piepho 2011). The data were considered homoscedastic when the residuals were randomly scattered around zero and the red line representing the mean of the residuals was horizontal and centered on zero in the residuals versus fitted plot. Similarly, the data were considered normally distributed when points lay close to the diagonal line, which represents the true normal quantiles, in the Q–Q plot. The skewness level of the data was also checked using the *skewness* function of the moments package (Komsta and Novomestky 2022). For traits in which the data appeared to violate the assumptions of homoscedasticity and normality, data transformation was applied prior to performing the ANOVA tests.

Post hoc mean comparisons were conducted via Tukey's honestly significant difference (HSD) test, with the Tukey HSD function in R, at a significance level of *p* < 0.05. To present the results of Tukey's test in tables or bar plots, a data summary of each independent variable was first created via the *group_by* function of the dplyr (Wickham et al. 2023) and plotrix (Lemon 2006) R packages. A compact letter display (CLD) was then generated via the *multcompLetters4* function of the multicompview R package (Graves et al. 2023) to indicate significantly different means with different letters. The results in boxplots and line graphs were plotted via the *ggplot* function of the ggplot2 package (Wickham 2016).

A correlation analysis was conducted to determine the relationships between genetic diversity parameters of the populations (specifically, allelic richness, expected heterozygosity, and population‐specific *F*st, extracted from Abate et al. 2024) with physical seed quality, in vitro germination, acclimatization, and seedling growth traits of seeds collected from these populations. The results of the correlation analysis are displayed in a correlation plot, which includes both numerical and graphical representations of the correlation intensity, generated using the *corr_coef* function of the metan package (Olivoto and Lúcio 2020).

## 3 Results

### 3.1 Summary of Genetic Diversity Analysis

The genetic diversity of the *A. gracilior* populations is summarized in Table 2, summarized by population size and developmental stage. The results revealed a clear trend of decreasing genetic diversity as population size diminishes and populations transition from adult to progeny cohorts. This decline is more consistent and pronounced in the progeny cohorts. For instance, allelic richness (Ar) values for progeny cohorts drop from 1.252 in large populations to 1.231 in intermediate populations and 1.214 in small/isolated populations. Similarly, unbiased expected heterozygosity (uHe) values decreased from 0.074 in large progeny populations to 0.072 in intermediate and 0.066 in small/isolated populations. Large populations also exhibited higher mean numbers of private alleles (*N*_PA_) compared to intermediate and small populations. While fixation indices (*F*_IS_) were high across populations, they did not display a consistent pattern relative to population size or developmental stage. Conversely, population‐specific *F*st values showed an inverse relationship with He and Ar, increasing as population size decreased, particularly among progeny cohorts.

**TABLE 2:** Mean genetic diversity indices summarized by population type and developmental stage, based on 1820 SNP markers generated using the DArTseq platform from 183 sampled genotypes. The values of these indices for the individual populations, along with further details about the SNP markers, are available in the companion paper (Abate et al. 2024).

Population type	Developmental stage	*N*	*N*_A_	*N*_PA_	A_R_	H_O_	uH_E_	*F*_IS_	*F*_ST_
Large/intact	Adult	28	2385	45	1.260	0.025	0.077	0.556	0.010
	Progeny	29	2372	43	1.252	0.028	0.074	0.511	0.017
	Mean		2378.5	44	1.256	0.027	0.075	0.534	0.014
Intermediate	Adult	27	2314	28	1.236	0.020	0.073	0.622	0.037
	Progeny	27	2305	28	1.231	0.024	0.072	0.563	0.046
	Mean		2310	28	1.233	0.022	0.072	0.593	0.041
Small/isolated	Adult	24	2302	36	1.234	0.023	0.074	0.578	0.005
	Progeny	28	2280	26	1.214	0.028	0.066	0.585	0.115
	Mean		2291	31	1.224	0.026	0.070	0.582	0.060

Abbreviations: A_R_: mean allelic richness; *F*_IS_: Fixation index (inbreeding coefficient); *F*_ST_: population‐specific *F*st; Ho: observed heterozygosity; *N*: number of individuals in the population that remained after data filtering; *N*_A_: total number of alleles; *N*_PA_: number of private alleles; uH_E_: unbiased expected heterozygosity.

### 3.2 Physical Seed Quality Characteristics

**FIGURE 6:**
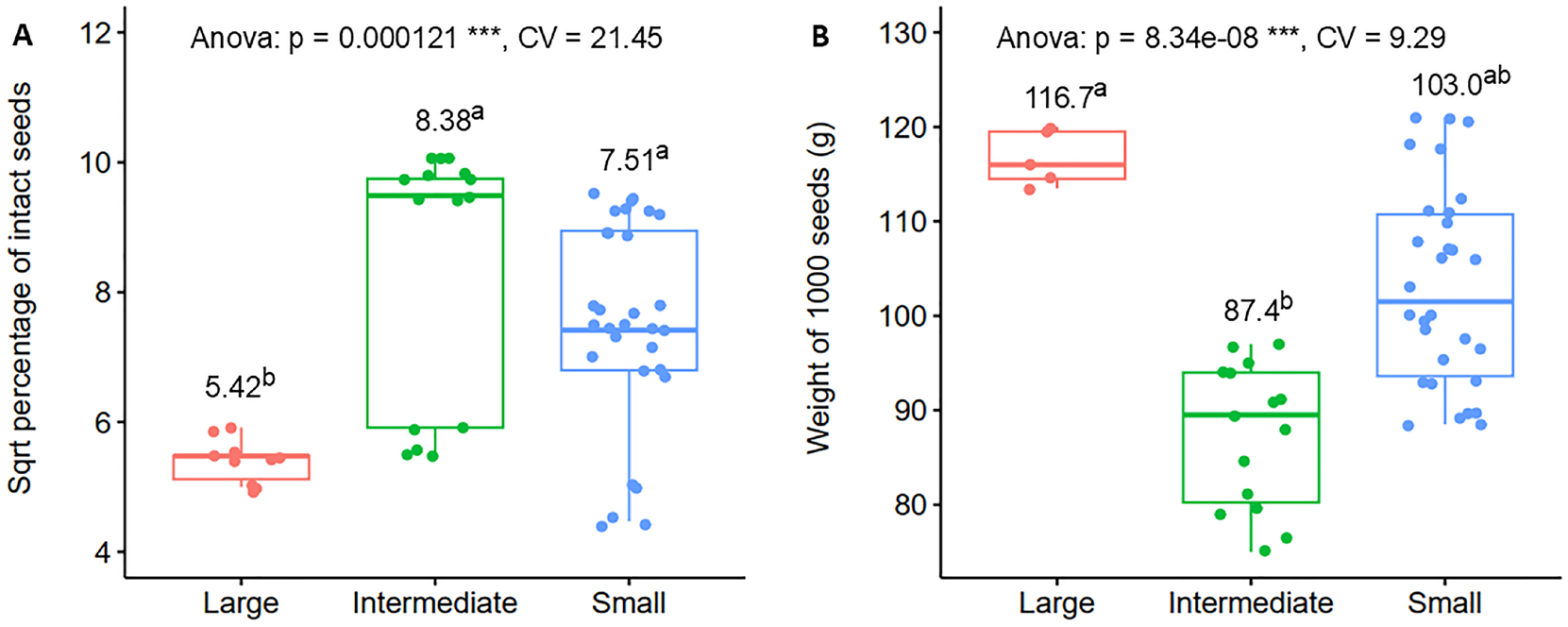
Physical seed quality comparison of *A. gracilior* seeds from different populations, grouped by population size categories: (A) Percent of intact seeds by population size class. (B) Weight of 100 seeds by population size class. The *p*‐values and coefficients of variation (CV) from the ANOVA tests are provided at the top of the plots; *** indicates significance at *p* ≤ 0.001. Mean values for each population size class are shown above the boxplots, accompanied by letters representing Tukey's HSD mean separation (*p* = 0.05).

ANOVA revealed significant variations (*p* < 0.001) among population size categories for all physical seed quality traits, such as the percentage of intact seeds and the weight of 1000 seeds (Appendices S1a,b and S2). Figure 6 presents boxplots comparing physical seed quality traits among population size classes. The intermediate population size class exhibited the highest percentage of intact seeds (sqrt‐transformed mean of 8.38), whereas the large population size class showed the lowest percentage, with a sqrt‐transformed mean of 5.42 (Figure 6A). For the weight of 1000 seeds, the large population size class had the highest score (mean of 116.7 g), while the intermediate class had the lowest score, with a mean of 87.4 g (Figure 6B).

### 3.3 *In Vitro* Germination

**FIGURE 7:**
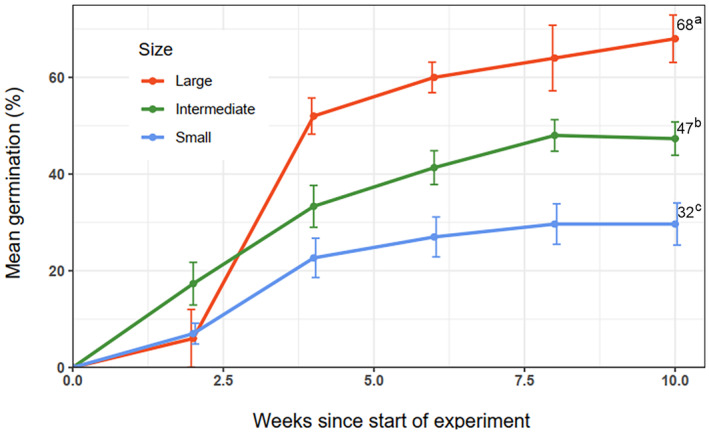
*In vitro* germination trend of *A. gracilior* seeds grouped by population size over a 10‐week period. The error bars indicate the means ± SE; the numbers and letters at the ends of the lines indicate Tukey's HSD mean separation of the final in vitro germination at week 10 (*p* = 0.05).

The results of the ANOVA for in vitro germination revealed significant differences (*p* < 0.001) among the population size classes (Appendices S1c and S2). Figure 7 shows the germination trend over the 10‐week in vitro test period, comparing the three population size categories throughout the germination period and at the end, which is typically considered the final germination percentage. The ‘large’ group had the highest germination scores, especially from the third week onward, with a mean final germination score of 68%. In contrast, the ‘small’ group consistently presented the lowest scores, with a mean final germination of 32%.

### 3.4 Acclimatization and Growth in a Lathhouse

**FIGURE 8:**
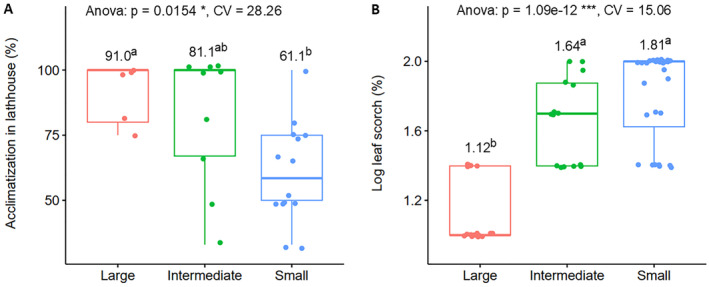
Acclimatization in the lathhouse and susceptibility to heat stress (leaf scorch scores) of in vitro germinated *A. gracilior* seedlings. (A) Survival of transferred seedlings after a 1‐month stay in the lathhouse, grouped by population size class and (B) leaf scorch at the end of the 8‐month experiment, grouped by population size classes. The *p*‐values and CV from the ANOVA tests are provided at the top of the plots; * and *** indicate significance at p ≤ 0.05 and *p* ≤ 0.001, respectively. Mean values for each population size class are shown above the boxplots, accompanied by letters representing Tukey's HSD mean separation (*p* = 0.05).

**FIGURE 9:**
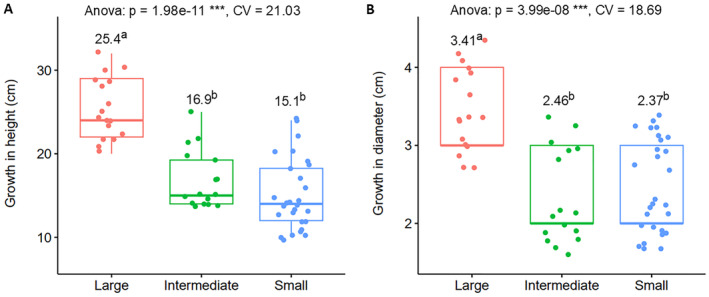
Growth performance of seedlings 8 months after the in vitro germinated *A. gracilior* seedlings were transferred to the lathhouse. (A) Height growth, grouped by population size classes, and (B) diameter growth, grouped by population size classes. The *p*‐values and CV from the ANOVA tests are provided at the top of the plots; *** indicates significance at *p* ≤ 0.001. Mean values for each population size class are shown above the boxplots, accompanied by letters representing Tukey's HSD mean separation (*p* = 0.05).

The results of the ANOVA (Appendices S1d–g and S2) revealed significant differences in acclimatization (*p* < 0.05), leaf scorch (*p* < 0.001), and growth traits (*p* < 0.001) among the population size classes. Figure 8 shows boxplots comparing the population size classes with respect to acclimatization and leaf scorch, which could be taken as measures of survival in the lathhouse after 1 month and at the end of the experiment, respectively. The ‘large’ population size group had the highest acclimatization score (mean of 91%) and lowest leaf scorch score (log‐transformed mean of 1.12), whereas the ‘small’ population size group had the lowest acclimatization score (mean of 61%) and highest leaf scorch score (log‐transformed mean of 1.81). The ‘intermediate’ group had acclimatization scores comparable to both the ‘large’ and ‘small’ groups, while its leaf scorch scores were comparable to the ‘small’ group. In Figure 9, a comparison of population size classes regarding seedling growth in terms of diameter and height in the lathhouse is presented. The ‘large’ population size class exhibited the highest seedling growth scores for both diameter (mean of 3.41 cm) and height (mean of 25.4 cm). Conversely, both the ‘intermediate’ and ‘small’ population categories had significantly lower growth performance compared to the ‘large’ category in terms of both height and diameter.

### 3.5 Relationships Between Genetic Diversity and Fitness Traits

**FIGURE 10:**
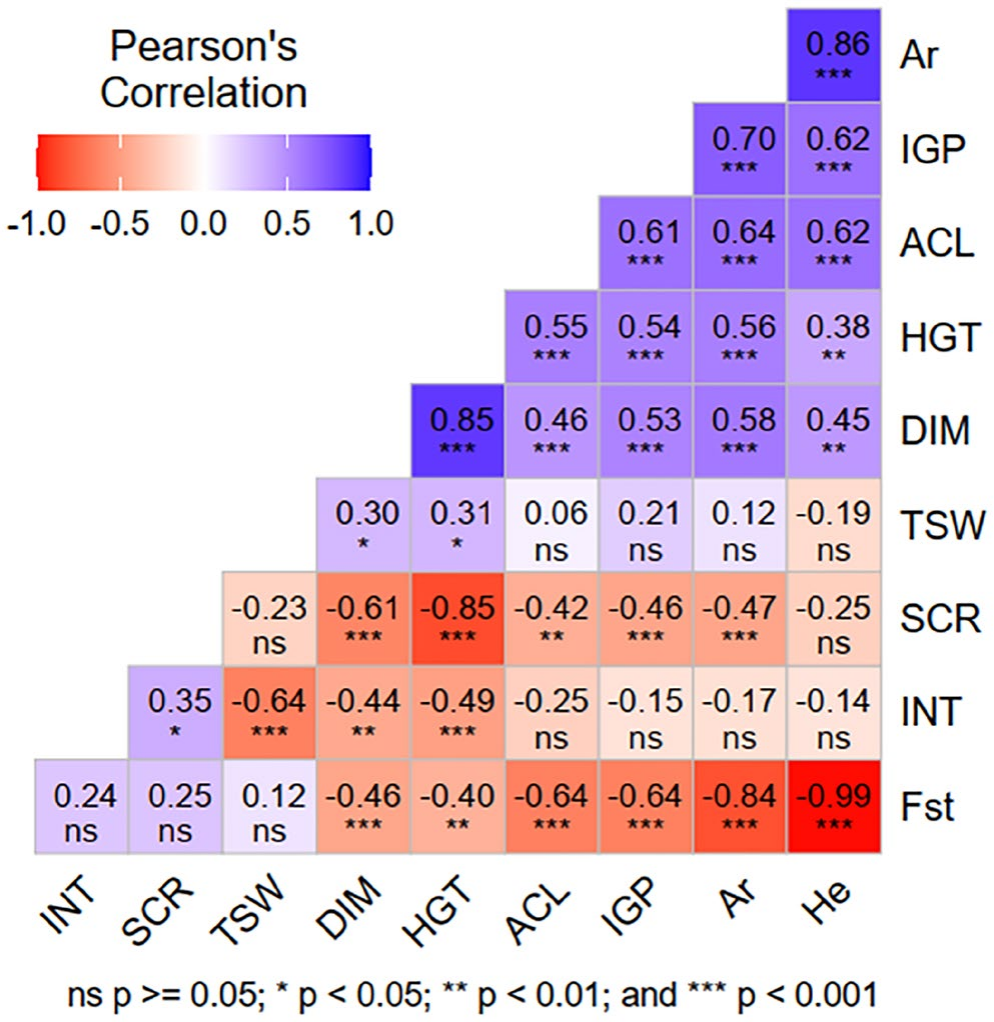
Correlations among genetic diversity, physical seed quality, in vitro germination, survival, and growth traits of *A. gracilior* seeds and seedlings from different populations. Each square displays Pearson's correlation coefficient, along with the corresponding level of significance, for the respective trait pairs. Positive correlations are shaded blue, while negative correlations are shaded red, with color intensity reflecting the strength of the correlation. ACL = acclimatization; Ar = allelic richness; DIM = diameter; *F*st = population‐specific *F*st; He = expected heterozygosity; HGT = height; IGP = *in vitro* germination percentage; INT = percentage of intact seeds; SCR = leaf scorch; TSW = weight of 1000 seeds.

Figure 10 displays the correlations between genetic diversity, physical seed quality, in vitro germination, acclimatization, and seedling growth traits. In accordance with some suggested guidelines (Mukaka 2012; Schober, Boer, and Schwarte 2018), the correlation coefficients were interpreted as follows: negligible (0–0.1), weak (0.1–0.39), moderate (0.4–0.69), strong (0.7–0.89), and very strong (0.9–1.0).

Notably, highly significant (*p* < 0.001), moderate to strong correlations were observed between the genetic diversity parameters (Ar and He) and in vitro germination, acclimatization, and seedling growth in diameter and height. The population‐specific *F*st also showed moderate to strong negative correlations with these germination and early progeny performance parameters. Seed weight had weak correlations with in vitro germination, acclimatization, and seedling growth. Seed intactness had a moderate negative correlation with seedling growth. Leaf scorch was negatively correlated with most other traits. *In vitro* germination showed a moderate positive correlation with acclimatization and seedling growth and a moderate negative correlation with leaf scorch.

## 4 Discussion

### 4.1 Genetic Diversity

Results of the genetic diversity analysis revealed a loss of genetic diversity (genetic erosion) in small or isolated populations of *A. gracilior* compared to relatively large, intact populations across fragmented landscapes. This decline was more pronounced along life‐stage transitions from adults to progeny. The reduction in genetic diversity was reflected in terms decreased allelic richness (Ar) and unbiased diversity, as well as an increase in population‐specific *F*st, an indicator of inbreeding and genetic drift (Table 2). Both Ar and uHe were adversely impacted by fragmentation, even though a recent meta‐analysis (González et al. 2020) indicates that self‐incompatible, long‐lived trees tend to lose allelic richness more rapidly than heterozygosity following fragmentation. This is explained by the faster loss of rare alleles that affects Ar, whereas changes in heterozygosity require more time to manifest in long‐lived organisms like trees (Guidugli et al. 2016; González et al. 2020). The simultaneous decline of both Ar and uHe across population sizes and life stages in the present study, despite *A. gracilior* being dioecious and long‐lived, may reflect the long time elapsed since fragmentation events, given the long history of forest disturbance in Ethiopia (Teketay 1992; Darbyshire, Lamb, and Umer 2003), and overlapping generations in adult populations. In support of this, the companion paper (Abate et al. 2024), based on phylogenetic analysis, attributed the overall low genetic diversity in the studied *A. gracilior* populations to a likely founder effect, where populations were suspected to have descended from ancestral populations that persisted in small numbers for generations.

Another notable result of the genetic diversity analysis is that while the fixation indices (*F*_IS_) scores were high across populations, indicating the presence of inbreeding, they did not follow a consistent pattern relative to population size or developmental stage, unlike Ar and uHe. This is consistent with the findings of a review article (Schlaepfer et al. 2018), which reported that *F*_IS_ is typically the least sensitive genetic diversity parameter to fragmentation. Moreover, Kardos et al. (2016) caution against relying solely on *F*_IS_ as a measure of inbreeding, as it can be biased by factors such as population size and allele frequency differences between sexes. In contrast, population‐specific *F*st values exhibited inverse patterns to He and Ar, increasing as population size decreased, particularly in progeny populations. This suggests that population‐specific *F*st may be a more robust indicator of inbreeding and genetic drift than *F*_IS_, providing insights into evolutionary divergence from an ancestral population (Weir and Goudet 2017; Kitada et al. 2021), making it a useful metric for measuring genetic drift.

The negative effects of forest fragmentation on the genetic diversity of *A. gracilior* observed in this study are consistent with findings in other wind‐pollinated and/or dioecious tree species, such as *Fagus sylvatica* (Jump and Peñuelas 2006), *Picea glauca* (O'Connell, Mosseler, and Rajora 2006), *Araucaria nemorosa* (Kettle et al. 2007, 2008), *Juniperus communis* (Provan et al. 2008), *Polylepis australis* (Seltmann et al. 2009), *Taxus baccata* (Dubreuil et al. 2010), *Spondias purpurea* (Cristóbal‐Pérez et al. 2021), and *Brosimum alicastrum* (Aguilar‐Aguilar et al. 2023). These studies consistently report consequences such as restricted gene flow (O'Connell, Mosseler, and Rajora 2006; Provan et al. 2008; Cristóbal‐Pérez et al. 2021), heightened genetic structure or differentiation (Jump and Peñuelas 2006; Provan et al. 2008; Seltmann et al. 2009), reduced genetic diversity and bottlenecks (Jump and Peñuelas 2006; Kettle et al. 2007, 2008; Cristóbal‐Pérez et al. 2021), elevated inbreeding levels (Jump and Peñuelas 2006; Kettle et al. 2007; Dubreuil et al. 2010; Cristóbal‐Pérez et al. 2021), correlated paternity (Cristóbal‐Pérez et al. 2021; Aguilar‐Aguilar et al. 2023), and diminished reproductive success and progeny fitness (O'Connell, Mosseler, and Rajora 2006; Kettle et al. 2008; Seltmann et al. 2009; Aguilar‐Aguilar et al. 2023) in fragmented compared to continuous populations. The population genetics paradigm underpinning these impacts (Young, Boyle, and Brown 1996; Frankham 2005; Lowe et al. 2005; Bacles and Jump 2011; Aguilar et al. 2019) explains them as consequences of reduced population size and increased isolation, which lead to allele loss, increased genetic drift, and restricted gene flow. These changes also elevate (biparental) inbreeding, resulting in higher homozygosity and the expression of deleterious alleles, culminating in inbreeding depression. This, in turn, reduces fecundity, seedling recruitment, and survival, ultimately threatening the long‐term viability of fragmented populations.

Conversely, studies on other species such as *Swietenia humilis* (White, Boshier, and Powell 2002), *Fraxinus excelsior* (Bacles et al. 2005), *Araucaria angustifolia* (Bittencourt and Sebbenn 2007), *Sorbus domestica* (Kamm et al. 2009), *Gomortega keule* (Lander, Boshier, and Harris 2010), *Eucalyptus leucoxylon* and *Eucalyptus camaldulensis* (Otwell et al. 2010), *Dysoxylum malabaricum* (Ismail et al. 2012), *Allocasuarina verticillata* (Broadhurst 2015), *Cariniana estrellensis* (Guidugli et al. 2016), and *Quercus species* (Ashley 2021) demonstrated that fragmented populations can remain functionally connected through extensive gene flow despite spatial isolation. Some of these studies have even suggested that small, isolated populations can act as genetic buffers or stepping stones connecting broader populations (White, Boshier, and Powell 2002; Lander, Boshier, and Harris 2010). Intriguingly, others pointed out that isolated trees in agroforestry or fragmented landscapes often receive pollen from genetically diverse, unrelated trees in multiple directions, serving as valuable sources of outbred seeds for forest restoration efforts (Ottewell et al. 2010; Ismail et al. 2012). These findings challenged theoretical expectations of population genetics, leading to the framing of forest fragmentation genetics as a “paradox” (Kramer et al. 2008).

This apparent resilience of trees to fragmentation has been attributed to mechanisms such as extensive pollen and seed dispersal, long lifespans, overlapping generations, and flexible mating systems (Kramer et al. 2008; Dubreuil et al. 2010; Lowe et al. 2015). However, more recent insights (Bacles and Jump 2011; Lowe et al. 2015; Aguilar et al. 2019; González et al. 2020) emphasize that not all tree species are equally immune to the genetic consequences of fragmentation. The degree of resilience or susceptibility varies depending on factors such as differences in mating systems (e.g., monoecy vs. dioecy, selfing vs. outcrossing), pollination mechanisms (wind vs. animal), pollinator mobility and specialization, seed dispersal modes, and the time elapsed since fragmentation occurred. In the present study, the long history of forest disturbance in Ethiopia, combined with the dioecy in *A. gracilior* and a potential founder effect (explained in Abate et al. 2024), likely contributed to the observed low genetic diversity and inbreeding depression in progeny from small/fragmented populations. These findings underline the necessity of species‐specific approaches when evaluating genetic responses to habitat fragmentation.

### 4.2 Reproduction and Early Progeny Vigor

Our study revealed significant variation (*p* < 0.05) among individual populations in terms of physical seed quality, in vitro germination, and survival and growth traits in the lath house. Notably, the “large” population size class consistently performed better (*p* < 0.05) across all the traits, except for the percentage of intact seeds, than did the smaller size classes. This disparity is likely due to the impact of inbreeding depression, which tends to be more pronounced in smaller and more fragmented populations than in larger, more intact populations. Consistent with this, the genetic diversity analyses revealed lower genetic diversity and greater inbreeding and genetic drift in the progeny from smaller populations (Table 2 and discussion in Section 4.1). Furthermore, the genetic diversity indices, specifically Ar, uHe and *F*st, exhibited strong positive correlations with the progeny fitness traits assessed in this study (Figure 10).

The observation of a lower percentage of intact seeds, alongside a greater proportion of empty and aborted seeds in larger populations, is contrary to our initial expectations. We anticipate that smaller, more isolated populations would exhibit greater inbreeding depression, leading to reduced fertilization and seed set and, consequently, a greater incidence of empty cones (parthenocarpy). Indeed, the genetic diversity analyses of the present study revealed lower genetic diversity and greater inbreeding in these small, isolated populations, supporting this expectation.

Fragmented populations often experience limited seed sets due to pollen limitation from increased isolation between mating individuals and embryo abortion from inbreeding depression (Offord et al. 1999; Kettle et al. 2008; Ahlinder, Giles, and García‐Gil 2021). Consistent with this, higher percentages of empty seeds in small and fragmented populations than in larger populations have been reported in other conifers, such as *Pinus strobus* (Rajora, Mosseler, and Major 2002), *Picea glauca* (O'Connell, Mosseler, and Rajora 2006), and *Araucaria nemorosa* (Kettle et al. 2008). Despite these findings, our seed quality assessment results appear to contradict both these reports and our own genetic diversity analysis. This discrepancy suggests that while inbreeding depression is expected to affect seed set, its impact might not be evident at the seed setting stage for *A. gracilior* . Inbreeding depression can manifest at various life stages, potentially influencing seed set and germination early on or becoming apparent later during growth and adult survival (Husband and Schemske 1996; Hill, Myra, and Johnston 2006; Ahlinder, Giles, and García‐Gil 2021). Consequently, the effects of inbreeding depression in *A. gracilior* may only become apparent in later developmental stages.

The lower percentage of intact seeds in larger populations observed in our study may be due to pollen movement being obstructed by the dense canopy of surrounding trees, leading to pollen limitation. Previous studies have demonstrated that plant density and the presence of other tree species in mixed forests can impact wind pollination by reducing the wind velocity and impeding pollen flow, which in turn decreases the effective dispersal distance of pollen (Whitehead 1969; Bittencourt and Sebbenn 2007; Hardy 2009; Millerón et al. 2012; Piotti et al. 2012). In this study, it appears that the dense canopy in larger populations hinders pollen flow, potentially reducing the amount of pollen reaching the mother trees. Despite this obstruction, larger populations seem to benefit from a more diverse range of pollen than do smaller and isolated populations. In contrast, mother trees in small and isolated populations may receive a quantity of pollen but experience reduced genetic diversity due to the limited number of mating individuals, leading to inbreeding. These observations align with the genetic diversity analysis and progeny fitness traits assessed in this study.

In the present study, seed weight did not appear to be influenced by inbreeding. Although the ‘large’ population size class exhibited the highest 1000‐seed weight, the lowest value was unexpectedly observed in the ‘intermediate’ class. If inbreeding was a significant factor, the lowest seed weight would be expected in the ‘small’ class. This suggests that other factors may have contributed to the observed variation in seed weight. During data collection, we noted that the soil in the two ‘intermediate’ populations (Borite and Halo in Table 1) was shallow and located on rocky outcrops. This indicates that environmental factors, particularly soil resource limitations, likely played a role in reducing seed weight in these populations. This finding aligns with O'Connell et al. (2006), who noted that environmental resource limitations can obscure the impact of inbreeding on seed weight and suggested correcting for this by dividing it by cone mass.

Another important observation is that seed production was greater in isolated trees and small populations with open canopies than in large populations with closed canopies (as described in Table 1). This is likely due to the full exposure of the crown to sunlight, which enhances seed production. These results, along with the data on intact, empty, and aborted seeds mentioned earlier, suggest that inbreeding depression resulting from habitat fragmentation may not be evident during the seed‐setting stage in *A. gracilior* .

There is no consistent trend in the literature regarding the effect of inbreeding on seed weight. Negative impacts of inbreeding on seed weight have been reported in species such as *Plantago coronopus* (Koelewijn and Van Damme 2005), *Shorea acuminata* (Naito et al. 2008), *Hymenaea courbaril* (Pereira et al. 2020), and *Pinus massoniana* (Wei et al. 2024). Conversely, in species such as *Silene latifolia* (Teixeira, Foerster, and Bernasconi 2009), *Pinus sylvestris* (Mullin et al. 2019), and *Pinus yunnanensis* (Li et al. 2024), inbreeding did not significantly affect seed weight. Additionally, Baskin and Baskin (2015) reported, in a review of 216 case studies, that the mean mass of inbred seeds was greater than that of outbred seeds in 12.5% of cases, equal in 38% of cases, and less in 49% of cases. In some cases, there may be a tradeoff between seed size and number, where pollen‐limited females produce fewer but larger seeds that perform better during germination to compensate for reduced seed production (Labouche, Richards, and Pannell 2016). When inbreeding depression is not apparent in seed weight, it may be due to the purging of inbred individuals early during the seed development stage (O'Connell, Mosseler, and Rajora 2006; Hill, Myra, and Johnston 2006; Li et al. 2024).

The significantly greater in vitro germination observed in the ‘large’ population suggests that inbreeding depression has impacted the growth and development of *A. gracilior* at this life stage. The absence of a strong correlation between in vitro germination and both seed intactness and seed weight further supports the idea that the differences in these traits between populations were not caused by inbreeding. This implies that the larger values of these traits do not represent genetic gains that would translate into enhanced progeny vigor in terms of germination, growth, and survival. Moreover, the ‘large’ population had significantly greater values for lath house survival and seedling growth traits, along with significantly lower values for leaf scorch, indicating that the effects of inbreeding depression also extend to later stages of survival and growth. This assertion is further reinforced by the strong positive correlations observed between genetic diversity parameters (Ar, uHe, and *F*st) and the germination, survival, and growth metrics.

The carry‐over effect of inbreeding from early to later life stages may be attributed to genes influencing early development, which can have cumulative effects as cell lineages multiply, as well as to pleiotropy, where a single gene affects multiple traits or functions (Husband and Schemske 1996). This finding aligns with the general theoretical model that suggests that inbreeding depression manifests in later life stages for selfing species and in both early and later stages for self‐incompatible and dioecious species (Husband and Schemske 1996; Ahlinder, Giles, and García‐Gil 2021). Additionally, the significantly higher leaf scorch scores observed in the ‘small’ populations imply that the occasionally elevated temperatures in the lathhouse may have exacerbated the effects of inbreeding depression. This observation is consistent with the literature, indicating that inbreeding depression is more pronounced in stressful environments than in benign environments. Stress can increase the expression of deleterious recessive alleles, which may eventually lead to their purging (Armbruster and Reed 2005; Fox and Reed 2010; Sandner, Matthies, and Waller 2021).

### 4.3 Concluding Remarks and Management Implications

This study demonstrated that population fragmentation leads to inbreeding depression in *A. gracilior* . Progeny from fragmented populations, comprising isolated individuals or small groups, exhibited significantly reduced in vitro germination, growth, and survival compared to progeny from larger populations. These findings are consistent with the genetic diversity analyses, which revealed lower genetic diversity and increased genetic drift in fragmented populations. Notably, inbreeding depression was not evident during the seed‐setting stage, as there were no significant differences in seed weight or the percentage of intact seeds. This suggests that for *A. gracilior* , the impacts of inbreeding depression manifest predominantly during later life stages, particularly germination and recruitment.

While this study provides robust insights, certain limiting factors should be considered when interpreting the results. As the first molecular marker‐based investigation of genetic diversity in *A. gracilior* —and, to the best of our knowledge, the genus—there is no baseline for comparison, particularly regarding the exceptionally low genetic diversity observed. Additionally, the study did not include gene flow or parentage analyses, which could have provided a more detailed understanding of the genetic consequences of habitat fragmentation. Furthermore, due to the unequal availability of population types (large, intermediate, and small) across study sites, a fully nested experimental design (population type by site) was not feasible for assessing reproductive success and progeny vigor. This limitation restricted us from accounting for the magnitude of site differences (i.e., those not attributable to the genetic effects of population size) on the observed variation. Despite these caveats, the study uncovered significant and consistent differences among population sizes using both molecular markers and phenotypic traits on reproduction and early progeny vigor. Importantly, the strong correlations observed between genetic diversity parameters and phenotypic traits bolster the validity of our conclusions. As a pioneering investigation into the genetics of *A. gracilior* , we believe this work contributes to the broader understanding of fragmentation genetics and provides a foundation for evidence‐based strategies aimed at conserving and restoring this important species.

The implications of these findings for the conservation and management of *A. gracilior* are substantial, particularly in guiding seed procurement practices. Seeds from isolated trees or small populations should be avoided, even when these populations produce abundant seeds and physical assessments suggest high seed quality. Instead, restoration efforts should prioritize sourcing seeds from larger, genetically diverse *A. gracilior* populations. To facilitate this, it is essential for authorities to identify and designate these larger populations as seed sources and enforce regulations to ensure compliance. This recommendation may appear to contradict previous studies (Lander, Boshier, and Harris 2010; Otwell et al. 2010; Ismail et al. 2012), which highlighted the potential of isolated trees in fragmented landscapes to provide genetically diverse, outbred seeds due to pollination from unrelated trees across multiple directions. This perspective stems from claims that isolated and scattered trees, as well as small populations, function as stepping stones for pollinators and enhance genetic connectivity in fragmented landscapes (White, Boshier, and Powell 2002; Lander, Boshier, and Harris 2010). While this may apply to species that depend on insect and vertebrate pollinators, we could not verify whether similar mechanisms hold true for the wind‐pollinated *A. gracilior* , as the present study did not include gene flow or parentage analysis. Nevertheless, the results of genetic diversity analysis and progeny vigor assessments suggest otherwise, highlighting the need for cautious and species‐specific seed procurement approaches in forest restoration efforts.

While the findings of the present study suggest prioritizing larger natural forest remnants and sacred sites that harbor greater genetic diversity, all populations examined showed a notable presence of private alleles (Abate et al. 2024; Table 2). This indicates that each population uniquely contributes to the species' gene pool and, therefore, deserves conservation attention. From a conservation perspective, no population should be dismissed as too small or insignificant. Although direct seed collection from small populations or isolated trees to be used alone is not recommended, an alternative strategy involves collecting seeds from multiple such populations, mixing them, and growing them as seedlings in nurseries. In fact, such mixed‐source germplasm approaches have been advocated as effective for genetic rescue, aiding species that suffer from genetic impoverishment and inbreeding depression (Finger et al. 2011; St. Clair et al. 2020). This strategy could enhance genetic diversity and bolster population resilience in restoration initiatives for *A. gracilior* .

## Author Contributions

**Nigussu Begashaw Abate:** conceptualization (lead), data curation (equal), formal analysis (equal), funding acquisition (equal), investigation (lead), methodology (equal), project administration (equal), visualization (equal), writing – original draft (lead). **Hewan Demissie Degu:** data curation (equal), formal analysis (equal), funding acquisition (equal), project administration (supporting), supervision (equal), validation (equal), visualization (equal), writing – review and editing (equal). **Marie Kalousová:** data curation (equal), formal analysis (equal), funding acquisition (equal), methodology (equal), supervision (supporting), visualization (equal), writing – review and editing (equal). **Tesfaye Abebe:** conceptualization (equal), funding acquisition (equal), project administration (lead), supervision (equal), writing – review and editing (equal).

## Conflicts of Interest

The authors declare no conflicts of interest.

## Supporting information


Appendix S1.

## Data Availability

All data used to generate the results in this manuscript can be accessed from the Dryad open online data repository: https://doi.org/10.5061/dryad.66t1g1kb7.
