# Supplementary material for: Inbreeding Depression Manifested in Progeny From Fragmented Populations of the Wind‐Pollinated Dioecious Conifer Afrocarpus gracilior (Pilg.) C. N. Page
Source: Ecol Evol. 2025 Feb 12;15(2):e70903. doi: 10.1002/ece3.70903 (PMC11815481; doi:10.1002/ece3.70903)
Supplement: Supplementary file 1 — Appendix S1. [file ECE3-15-e70903-s001.docx]

**Appendix 1**

**ANOVA models and outputs**

1. Percent of intact seeds by population size


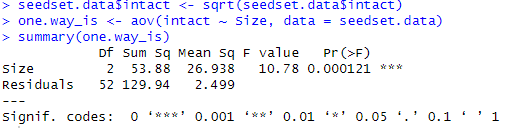


1. Seed weight by population size


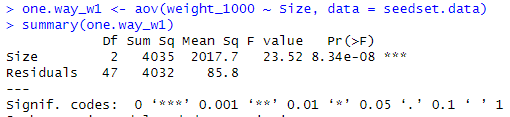


1. *In vitro* germination by population size


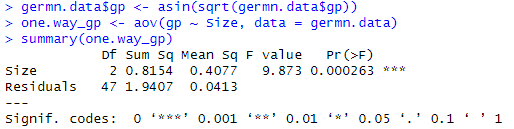


1. Lathhouse acclimatization by population size


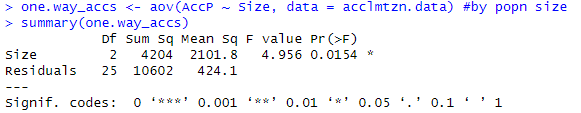


1. Growth in height by population size


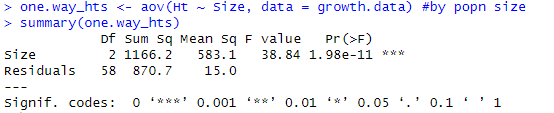


1. Growth in diameter by population size


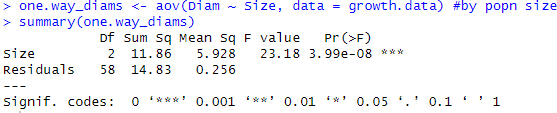


1. Leaf scorch by population size


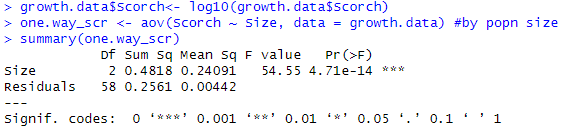


**Appendix 2**

**Tukey’s HSD post-hoc mean separation of traits used in the ANOVA.** Abbreviations: INT = percentage of intact seeds (data square root transformed); TSW = weight of 1000 seeds; IGP = *in vitro* germination percentage (data arcsine transformed); ACL = acclimatization; DIM = diameter; HGT = height; SCR = leaf scorch (data log_10_ transformed).

| Population size class | INT (sqrt) | TSW | IGP  (asin) | ACL | HGT | DIM | SCR (log) |
| --- | --- | --- | --- | --- | --- | --- | --- |
| Large | 8.4±2.0^a^ | 116.7±2.9^a^ | 0.97±0.12^a^ | 91±5.6^a^ | 25.4±3.6^a^ | 3.4±0.5^a^ | 1.8±0.3^a^ |
| Intermediate | 7.5±1.6^a^ | 103±10.5^b^ | 0.76±0.14^b^ | 81.1±8.6^ab^ | 16.9±3.4^b^ | 2.5±0.5^b^ | 1.6±0.2^a^ |
| Small | 5.4±0.3^b^ | 87.4±7.6^b^ | 0.58±0.24^c^ | 61.1±5.1^b^ | 15.1±2.2^b^ | 2.4±0.5^b^ | 1.1±0.2^b^ |
